# Resection of primary cardiac angiosarcoma infiltrating the right atrioventricular junction and tricuspid valve: a case report

**DOI:** 10.1186/s13019-021-01426-w

**Published:** 2021-03-21

**Authors:** Lubna Bakr, Hussam AlKhalaf, Ahmad Takriti

**Affiliations:** Department of Cardiac Surgery, Faculty of Medicine, Damascus University, Damascus, Syria

**Keywords:** Primary angiosarcoma, Cardiac sarcoma, Sarcoma of the heart, Infiltration, Heterologous pericardium, Reconstruction, Chemotherapy, Radiotherapy, Survival, Case report

## Abstract

**Background:**

Primary cardiac tumours are extremely rare. Most of them are benign. Sarcomas account for 95% of the malignant tumours. Prognosis of primary cardiac angiosarcoma remains poor. Complete surgical resection is oftentimes hampered when there is extensive tumour involvement into important cardiac apparatus. We report a case of cardiac angiosarcoma of the right atrium and ventricle, infiltrating the right atrioventricular junction and tricuspid valve.

**Case presentation:**

Initially, a 22-year-old man presented with dyspnoea. One year later, he had recurrent pericardial effusion. Afterwards, echocardiography revealed a large mass in the right atrium, expanding from the roof of the right atrium to the tricuspid valve. The mass was causing compression on the tricuspid valve, and another mass was seen in the right ventricle. Complete resection of the tumour was impossible. The mass was resected with the biggest possible margins. The right atrium was reconstructed using heterologous pericardium. The patient’s postoperative course was uneventful. Postoperative echocardiography showed a small mass remaining in the right side of the heart. Histopathology and immunohistochemistry confirmed the diagnosis of angiosarcoma. The patient underwent adjuvant chemotherapy and radiotherapy later on. He survived for 1 year and 5 days after the surgery. After a diagnosis of lung and brain metastases, he ended up on mechanical ventilation for 48 h and died.

**Conclusions:**

Surgical resection combined with postoperative chemotherapy and radiotherapy is feasible even in patients with an advanced stage of cardiac angiosarcoma when it is impossible to perform complete surgical resection.

## Background

Primary cardiac tumours are extremely rare. Most primary cardiac tumours are benign, and around one quarter is malignant. Sarcomas account for 95% of these malignant tumours [1]. Prognosis of primary cardiac angiosarcoma remains poor [2]. Complete surgical resection is the most critical factor to achieve better long-term outcome in treating primary cardiac sarcomas, however, it is oftentimes hampered when there is extensive tumour involvement into important cardiac apparatus [3]. We report a case of cardiac angiosarcoma of the right atrium and ventricle, infiltrating the right atrioventricular junction and tricuspid valve.

## Case presentation

Initially, a 22-year-old man presented with dyspnoea. He was prescribed some sprays by a pneumologist. One year later, pericarditis was suspected. Transthoracic echocardiography showed pericardial effusion, and pericardiocentesis was performed. The cardiologist prescribed colchicine, but pericardial effusion came back.

The young man was referred to an echocardiogram expert, and transthoracic echocardiography (TTE) revealed a large hypoechoic mass (7.0 × 4.3 cm) with a broad base in the right atrium, expanding from the roof of the right atrium to the tricuspid valve. The tricuspid flow was normal, and a widened inferior vena cava was seen. After admission, transoesophageal echocardiography (TEE) confirmed the same findings, however it elucidated that the mass was causing compression on the tricuspid valve. It also revealed another mass in the right ventricle.

**Fig. 1:**
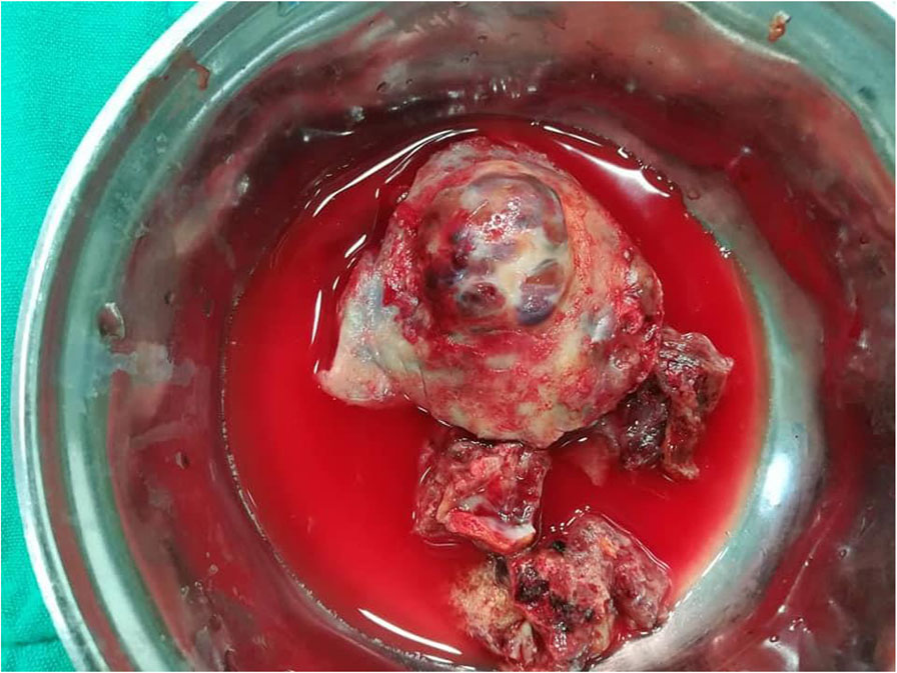
The mass after surgical resection was performed

**Fig. 2:**
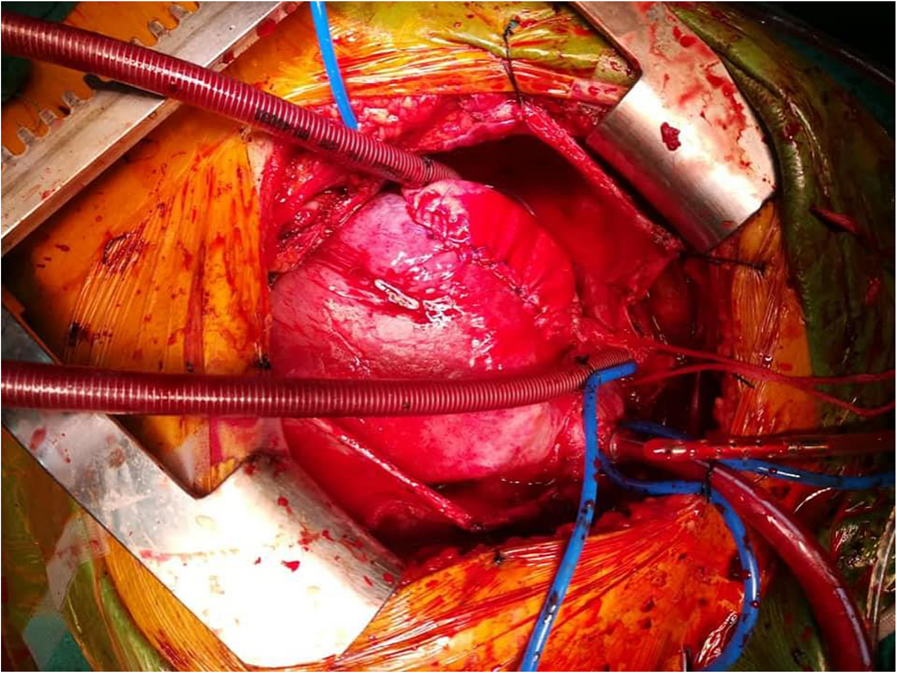
Reconstruction of the right atrium using a patch of heterologous pericardium

The surgical procedure was a week later. A median sternotomy was performed, followed by a pericardiotomy. The large mass was seen in the right atrial wall. Cardiopulmonary bypass was initiated, and the heart was arrested. Complete resection of the tumour was impossible. The mass was resected with the biggest possible margins (Fig. 1). A narrow margin (1 cm) of the right atrium was left in place, so that reconstruction by suturing the pericardium patch could be possible. The tumour was found to be extensively infiltrating the right ventricle, the right atrioventricular junction and tricuspid valve. The right atrium was reconstructed using a patch of heterologous glutaraldehyde-preserved pericardium (Fig. 2).

The patient’s postoperative course was uneventful. He underwent postoperative TTE 6 days later. It revealed a small hypoechoic mass remaining in the right side of the heart (4.3 × 2.3 cm) compressing the tricuspid valve, but causing no stenosis. The young man was discharged home. Histopathology and immunohistochemistry confirmed the diagnosis of angiosarcoma. The patient was referred to oncology department for further management. He underwent adjuvant chemotherapy and radiotherapy later on. We kept in contact with the patient and his family despite them living in a remote area, and his parent reported his death 1 year and 5 days after the surgery. After a diagnosis of lung and brain metastases, he ended up on mechanical ventilation for 48 h and died.

## Discussion and conclusions

Resection of cardiac angiosarcoma has poor prognosis in the long run. However, it might be life-prolonging. Complete surgical resection is possible in less than 50% of patients, with a mean survival of 3 months to 1 year [1]. This young patient could not undergo complete surgical resection, and yet survived 1 year and 5 days after the surgical procedure, which makes the survival period in this case outstanding considering the fact that the disease was in an advanced stage.

The patient initially presented with dyspnoea, which was reported previously in other cases of cardiac angiosarcoma [4–6]. Pericardial effusion was seen in our case as in other reported cases [1, 2]. Cardiac angiosarcoma can infiltrate many parts of the heart. In this patient, it was seen in the right atrium and ventricle, and infiltrating the right atrioventricular junction and tricuspid valve. It had this type of infiltration in another reported case [3]. It was reported in other locations, including the right atrium [6, 7], the left atrium [4], the pericardium [7], the aorta [8], the ventricular outflow tract (RVOT) and main pulmonary artery (MPA) [5]. Angiosarcomas arising from saphenous vein femoropopliteal bypass grafts have also been reported [9]. Primary angiosarcoma can be a rare cause of RVOT obstruction [5]. However, this was not seen in our case.

Cardiac angiosarcomas are the most common primary differentiated cardiac neoplasms. However, the aetiology of these lesions is not known [10]. The genetics of primary cardiac tumours is poorly understood. Although several complexes with genetic links have been associated with benign primary cardiac myxomas, there are no demonstrable associations with malignant sarcomas [11]. No genetic testing was performed in our case.

Although there are currently no guidelines or effective therapeutic strategies [6], patients are usually treated by surgery when possible with adjuvant radiotherapy / chemotherapy [1]. Management of this case depended on the surgeon’s judgement. The right atrial free wall reconstruction was done using a patch of heterologous glutaraldehyde-preserved pericardium, which was previously reported in the literature [6]. Pedicled autologous pericardium was also reported to be used for reconstruction of the right atrial free wall and vena cava [2]. There was no way to be sure that the sutures would hold, as the tumour was infiltrating the whole atrial wall. A decision was made to conduct surgical resection as the patient had a very poor prognosis at the time of surgery. The call was made as a life-prolonging measure. After being discharged from the hospital, the patient underwent chemotherapy trying to avoid metastases. He underwent radiotherapy as well. As the patient lived in another part of the country, and due to difficult circumstances in Syria, further imaging studies could not be obtained. However, his parent was able to report the exact survival period after the operation which was 1 year and 5 days. Survival is not reported in all case reports of cardiac angiosarcoma. However, in one case the patient underwent surgery and died 12 months later because of multiple organ failure due to metastasis [2], which was similar to our case. A shorter survival period of 10 months after surgery was reported, due to impossible complete resection [6].

In conclusion, surgical resection combined with postoperative chemotherapy and radiotherapy is feasible even in patients with an advanced stage of cardiac angiosarcoma when it is impossible to perform complete surgical resection. However, the surgeon has always to emphasize that such cases have poor prognosis.

## Data Availability

The authors declare that the data supporting the findings of this study are available within the article and its supplementary information files.

## Abbreviations

TTE: Transthoracic echocardiography
TEE: Transoesophageal echocardiography
RVOT: Right ventricle outflow tract
MPA: Main pulmonary artery
